# Characterizing the Potential of the Non-Conventional Yeast *Saccharomycodes ludwigii* UTAD17 in Winemaking

**DOI:** 10.3390/microorganisms7110478

**Published:** 2019-10-23

**Authors:** Marcos Esteves, Catarina Barbosa, Isabel Vasconcelos, Maria João Tavares, Arlete Mendes-Faia, Nuno Pereira Mira, Ana Mendes-Ferreira

**Affiliations:** 1 WM&B—Laboratory of Wine Microbiology & Biotechnology, Department of Biology and Environment, Universidade de Trás-os-Montes e Alto Douro, 5000-801 Vila Real, Portugal; mjcesteves@utad.pt (M.E.); crbarbosa@utad.pt (C.B.); afaia@utad.pt (A.M.-F.); 2 BioISI—Biosystems & Integrative Sciences Institute, Campo Grande, 1749-016 Lisboa, Portugal; 3 CoLAB Vines&Wines - National Collaborative Laboratory for the Portuguese Wine Sector, Associação para o Desenvolvimento da Viticultura Duriense (ADVID), Edifício Centro de Excelência da Vinha e do Vinho, Régia Douro Park, 5000-033 Vila Real, Portugal; 4 CBQF/Centro de Biotecnologia e Química Fina, Escola Superior de Biotecnologia, Universidade Católica Portuguesa, 4169-005 Porto, Portugal; ivasconcelos@porto.ucp.pt; 5 iBB - Institute of Bioengineering and Biosciences, Department of Bioengineering, Instituto Superior Técnico, Universidade de Lisboa, 1049-001 Lisboa, Portugal; tavares.mja@gmail.com (M.J.T.); nuno.mira@tecnico.ulisboa.pt (N.P.M.)

**Keywords:** *Saccharomycodes ludwigii*, non-*Saccharomyces*, wine fermentation, mixed-culture, wine aroma

## Abstract

Non-*Saccharomyces* yeasts have received increased attention by researchers and winemakers, due to their particular contributions to the characteristics of wine. In this group, *Saccharomycodes ludwigii* is one of the less studied species. In the present study, a native *S. ludwigii* strain, UTAD17 isolated from the Douro wine region was characterized for relevant oenological traits. The genome of UTAD17 was recently sequenced. Its potential use in winemaking was further evaluated by conducting grape-juice fermentations, either in single or in mixed-cultures, with *Saccharomyces cerevisiae*, following two inoculation strategies (simultaneous and sequential). In a pure culture, *S. ludwigii* UTAD17 was able to ferment all sugars in a reasonable time without impairing the wine quality, producing low levels of acetic acid and ethyl acetate. The overall effects of *S. ludwigii* UTAD17 in a mixed-culture fermentation were highly dependent on the inoculation strategy which dictated the dominance of each yeast strain. Wines whose fermentation was governed by *S. ludwigii* UTAD17 presented low levels of secondary aroma compounds and were chemically distinct from those fermented by *S. cerevisiae*. Based on these results, a future use of this non-*Saccharomyces* yeast either in monoculture fermentations or as a co-starter culture with *S. cerevisiae* for the production of wines with greater expression of the grape varietal character and with flavor diversity could be foreseen.

## 1. Introduction

Inoculation with active dry yeasts of *Saccharomyces cerevisiae* is a common practice in most wine-producing regions since the middle of the 20th century, to assure prompt and reliable fermentations and wines with a consistent and predictable quality. The main drawback of this practice is the loss of the typical distinctive characteristics found in wines obtained by spontaneous fermentation, carried out by winery- and grape-resident yeasts [1,2,3,4,5]. Grape-must microbiota is dominated by non-*Saccharomyces* yeast strains which have for long been considered spoilage agents, not only due to their low fermentative ability but also because of the assumption that they overproduce off-flavor compounds, such as acetic acid, acetaldehyde, acetoin, or ethyl acetate [1,6]. However, the demonstration that these negative traits are species and strain-dependent [7] and that some non-*Saccharomyces* yeasts even exhibit beneficial traits, not found in *S. cerevisiae* [8,9,10,11,12,13,14] have led winemakers to take a fresh look at these formerly disregarded species. In this line, over the last years, a massive number of studies searching for autochthonous non-*Saccharomyces* strains that might impart a unique aroma complexity or mouthfeel to wines, while expressing terroir-associated characteristics have been published [15,16,17,18,19,20,21,22,23]. Among the most studied non-*Saccharomyces* yeasts are the members of the genus *Hanseniaspora* (*H. uvarum* [24,25,26,27,28], *H. guillermondii* [24,25,26,29,30], and *H. vineae* [25,31,32,33,34]), *Metschnikowia pulcherrima* [21,33,35,36,37], *Torulaspora delbrueckii* [38,39,40,41,42,43], *Kluyveromyces*/*Lachancea thermotolerans* [44,45,46,47,48], and *Starmerella bacillaris* (formerly *Candida stellata/Candida zemplinina*) [27,49,50,51,52,53]. In addition to their contribution to the enhancement and diversification of wine aroma, it was found that these yeasts might display other oenological relevant traits, such as increased glycerol, mannoprotein, and total acidity contents [16,46,52,54,55], contributing to color stability [56,57] and reducing volatile acidity or ethanol levels [21,33,36,38,58]. In this context, the potential of developing starter cultures based on non-*Saccharomyces* yeast species has flourished in the wine world and several non-*Saccharomyces* strains that can be used as starter cultures are now commercially available [10,59]. Nevertheless, there are still a number of species whose potential in winemaking remains to be discovered. One such species is *Saccharomycodes ludwigii* (*S. ludwigii*), a bipolar budding yeast, first isolated from deciduous trees in Europe [60], which has a long history as a spoilage agent in winemaking. This yeast is rarely found in grapes but appears to be a usual contaminant of sulfite-preserved musts [6]. It has also been found in wines, at the end of the alcoholic fermentation or during storage [61], where it contributes for sedimentation or cloudiness formation [62]. The persistence of *S. ludwigii* in wineries is largely explained by its high tolerance to sulfur dioxide [63] and ethanol [7]. Regardless of its association with spoilage, *S. ludwigii* has been proposed as a starter-culture for the production of feijoa fermented beverages [61], cider [64], and low-alcohol or non-alcoholic beers [65,66]. In winemaking, the few studies undertaken with this yeast have shown that it could also be promising, since, depending on the strain, *S. ludwigii* possesses a good fermentative capacity [7] and is able to shape the aroma profile [8,18] and mouthfeel perception of wines [52]. Thus, the aim of this work was to examine the oenological potential of *S. ludwigii* UTAD17, an indigenous Douro Wine Region strain whose genome sequence has been recently released [67]. For this purpose, besides phenotyping the strain for relevant oenological traits, fermentations of a natural grape-must were performed, either in pure or in co-culture with *S. cerevisiae.* The growth and fermentation behavior, as well as the analytical profiles of the final wines, were also evaluated, revealing that this strain could be useful for tailoring wines with enhanced varietal characters.

## 2. Materials and Methods 

### 2.1. Yeast Strains and Maintenance Conditions

The yeast *S. ludwigii* UTAD17, an autochthonous Douro Wine Region strain isolated in our laboratory [67], and the *S. cerevisiae* Lalvin QA23 (Lallemand-Proenol 4410-308 Canelas, Portugal), obtained from the market as an active dried yeast, were used in this study. Yeasts were routinely maintained at 4 °C on Yeast Extract Peptone Dextrose agar plates (YPD) containing per liter: 20 g of glucose, 10 g of peptone, 5 g of yeast extract, and 20 g of agar from stocks stored at −80 °C. Prior to use, the yeasts were transferred to a new slant of YPD and incubated for 24–48 h at 28 °C, unless otherwise stated.

### 2.2. Phenotypic Characterization

*S. ludwigii* UTAD17 was screened for relevant enological features [68]. The evaluation of stress resistance and the activity of the enzymes of enological interest was performed as described in [21]. Briefly, for all assays, after growth in YPD medium until the mid-exponential growth phase, the yeast strain was inoculated in the appropriate culture media for stress tolerance and enzymatic activities evaluation. YPD agar plates without a stress agent, was used as the control. Accordingly, the following concentrations were used—6%, 9%, or 12% (*v*:*v*^−1^) of ethanol; 1, 2, or 4 mM of sulfur dioxide (SO_2_); 0.5, 1, or 2 mM of copper, supplied as copper sulfate; and 0.25, 0.5, or 1 mM of H_2_O_2_. Yeast growth in the presence of cerulenin or 5,5′,5′′-trifluoro-d,l-leucine (TFL) was screened to evaluate the potential to produce particular flavor compounds, using agar plates and a minimal medium (YNB) supplemented with glucose (2%) and TFL (0.6 mM) or cerulenin (6 µM). 

Enzymatic activities were evaluated using qualitative assays. The activity of β-lyase was screened using a medium containing 0.1% *S*-methyl-l-cysteine, 0.01% pyridoxal-50-phosphate, 1.2% Yeast Carbon Base, and 2% agar, with pH adjusted to 3.5. β-glycosidase activity was tested using a medium containing 0.5% cellobiose (4-*O*-β-d-glucopyranosyl-d-glucose), 0.67% yeast nitrogen base, and 2% agar. Proteolytic activity was evaluated by spotting yeast strain on skim milk agar medium. Qualitative detection of biogenic amines (histamine, tyramine and putrescine) was performed using differential culture media containing yeast extract (3%), glucose (1%), the amino acid precursor (2%), histidine, tyrosine or ornithine, and bromocresol purple (0.015 g.L^−1^), at a final pH adjusted to 5.2. The potential ability to produce hydrogen sulfide (H_2_S) associated to sulfite reductase activity was evaluated by growing yeast cells on BiGGY agar.

### 2.3. Grape Juice and Inocula Preparation

Natural grape-juice was obtained by crushing grapes of the *Vitis vinifera* L. cv. Touriga Nacional; after homogenization, the juice was clarified by centrifugation at 12,734× *g* for 10 min (Sorvall centrifuge GSA 6-Place Rotor, Marshall Scientific, Hampton, NH 03842, USA) and was carefully separated from the solid fraction. A sample of the grape-juice was collected at this point for routine analysis (Table 1). After pasteurization at 70 °C for 10 min, the grape-juice was immediately cooled on ice. For each strain, the inoculum was prepared by separately pre-growing the yeast cells in 50 mL-flasks, containing 25 mL of synthetic grape-juice medium (GJM), original recipe of [69] with minor modifications in the nitrogen composition. Nitrogen was added up to 267 mg YAN/L, supplied as di-ammonium phosphate (DAP). The flasks were incubated overnight at 25 °C in an orbital shaker (IKA KS 4000 ic Control, VWR International, Radnor, PA 19087-8660, USA) set at 150 rpm.min^−1^. Both strains were inoculated in grape-juice with an initial cellular concentration of 10^6^ cfu·mL^−1^.

### 2.4. Fermentation Trials 

Fermentations trials were conducted by inoculating (1) a single culture of *S. ludwigii* UTAD17 (Sl), (2) a single culture of *S. cerevisiae* (Sc), (3) a mixed culture of *S. ludwigii* UTAD17 and *S. cerevisiae* (Sl+Sc) inoculated simultaneously, prior to fermentation, or (4) a mixed culture in which *S. cerevisiae* was inoculated sequentially, 72 h after *S. ludwigii* UTAD17 (Sl_Sc). Single and mixed culture fermentations were carried out in duplicates and triplicates, respectively, using a previously described system [70] consisting of 100 mL flasks filled to 2/3 of their volume (80 mL) and fitted with a side-arm port sealed with a rubber septum for anaerobic sampling. Two flasks containing uninoculated grape-must were used as control. The flasks were maintained at 25 °C under static conditions. Fermentations were monitored daily by weight loss as an estimation of CO_2_ production and were allowed to proceed until no further weight loss was observed. For the assessment of growth parameters and analytical determinations, aseptic sampling was performed using a syringe-type system. After fermentation, the wines were centrifuged (10 min at 5500 rpm, Sigma 3-18K refrigerated Centrifuge, 37520 Osterode am Harz, DE) to remove yeast cells and were kept at −20 °C until the analytical determinations were performed. 

### 2.5. Determination of Growth and Fermentation Parameters

Growth kinetics were monitored by viable cell plate counting (cfu·mL^−1^) on YPD agar or Lysine agar medium plates incubated at 28 °C for 48–72 h. The lysine agar medium was used to directly assess *S. ludwigii* UTAD17 viability in mixed-culture fermentations, since *S. cerevisiae* is unable to grow in a culture medium in which lysine is the sole nitrogen source [71]. The maximum fermentation rate (M_ax_FR) was determined from the slope of the linear dependence of the steepest incline in weight (g) at the corresponding time points (h), and fermentation purity (FP) was determined as acetic acid (g L^−1^)/ethanol (%*v*:*v*^−1^). 

### 2.6. Analytical Determinations

The amount of glucose and fructose, acetic acid, as well as Yeast Assimilable Nitrogen (YAN), comprising primary amino nitrogen (PAN) and ammonium, were enzymatically determined using a Y15 autoanalyzer (Biosystems S.A, Barcelona, Spain). Total SO_2_, pH, and titratable acidity were determined according to the standard methods compiled in the Compendium of International Methods of Analysis of Musts and Wines [72]. 

Ethanol and glycerol concentrations were determined in a high-performance liquid chromatography system (HPLC Flexar, PerkinElmer, Shelton, Connecticut, EE. UU) equipped with the ion exclusion cation exchange column Aminex HPX-87H (Bio-Rad Laboratories, Hercules, CA, USA) and refractive index detector. The column was eluted using sulfuric acid (0.005 N) at 60 °C and a 0.6 mL/min flow rate. Samples were previously filtered through a membrane (Millipore, 0.22 μm pore size) before an injection of 6 μL. The components were identified through their relative retention times, compared to the respective standards, using the Perkin Elmer Chromera Software.

Aliphatic higher alcohols (1-propanol, 1-butanol, 2-methyl-1-butanol and 3-methyl-1-butanol), acetaldehyde, and ethyl acetate were analyzed as described Moreira et al. [73] by using a Hewlett-Packard 5890 (Hewlett-Packard, Palo Alto, CA 94304, USA) gas chromatograph equipped with a flame ionization detector (GC-FID) and connected to a H.P. 3396 Integrator. Fifty microliters of 4-methyl-2- pentanol at 10 g L^−1^ was added to 5 mL of wine as the internal standard. The sample (1 μL) was injected (split, 1: 30) into a CP-WAX 57 CB column (Chrompack) of 50 m × 0.25 mm and 0.2 μm phase thickness. The program temperature varied from 40 °C (10 min) to 80 °C (10 min) at 3 °C min^−1^ and from 80 °C to 200 °C (4 min) at 15 °C min^−1^. Injector and detector temperatures were set at 220 °C. Carrier gas was H_2_ at 1–2 mL min^−1^. 

The determination of 2-phenylethanol, acetates of higher alcohols (isoamyl acetate, 2-phenylethyl acetate) and ethyl esters of fatty acids (ethyl butanoate, ethyl hexanoate and ethyl octanoate), volatile fatty acids (butyric, isobutyric, isovaleric acids) and free fatty acids (hexanoic, octanoic and decanoic acids) was performed in a Hewlett Packard 5890 gas chromatograph, equipped with a flame ionization detector. For this purpose, 50 mL of wine, with 4-decanol at 1.5 mg/L as the internal standard, was extracted successively with 4, 2, and 2 mL of ether–hexane (1:1 *v*:*v*^−1^) for 5 min. The organic phase (1 μL) was injected (splitless) into a BP21 (SGE) column of 50 m × 0.22 mm and 0.25 μm phase thickness. The temperature program was 40 °C (1 min) to 220 °C (15 min), at 2 °C·min^−1^. Injector and detector temperatures were set at 220 °C. The carrier gas used was H_2_ at 1–2 mL min^−1^.

### 2.7. Statistical Analysis 

The data are presented as mean values with their standard deviation. One-way analysis of variance (ANOVA) of the inoculation strategy on yeast growth, fermentation activity, and volatile and non-volatile compounds was performed using the JMP 7.0 software (SAS Inc., 2007). If significant differences were found with ANOVA (*p* < 0.05), then Student’s *t*-test was used for the paired comparisons. Partial least squares linear discriminant analysis (PLS–DA) was performed to discriminate the wines, based on volatile and non-volatile compounds, using the MATLAB R2018b environment (The MathWorks Inc.; version 9.5.; Natick, MA, USA) state abbreviation). All data were previously standardized.

### 2.8. Comparison of S. ludwigii UTAD17 ‘ORFeome’ with S. cerevisiae s288c

Recently *S. ludwigii* UTAD17 has been sequenced and annotated with the predicted set of open reading frames (ORFeome) estimated to be about 4015 protein-coding genes [67]. This whole-genome shotgun is available in the European Nucleotide Archive (ENA) under the accession number UFAJ01000000 (contigs UFAJ01000001 through UFAJ01001360; study accession number PRJEB27462; read accession number SAMEA4945973). Herein, a supervised analysis was performed by BLASTp using the proteomes of *S. ludwigii* UTAD17 and *S. cerevisiae* S288c, looking for the presence or absence of protein-coding genes that could underlie the observed physiological traits. *S. ludwigii* UTAD17 proteins were considered similar to those present in *S. cerevisiae* S288c when the resulting alignment had an associated e-value below e^−20^ and a minimum identity of 30% (Table S1). 

## 3. Results and Discussion

### 3.1. Phenotypic Characterization of S. ludwigii UTAD17 

In order to assess the potential of *S. ludwigii* UTAD17 to be used in winemaking, a phenotypic profiling was performed for a number of oenological traits, as determined by the International Organisation of Vine and Wine (OIV) [68] (Figure S1). Ethanol is the main metabolite produced during wine fermentation while SO_2_ and copper are applied by winemakers as antimicrobial agents to control spoilage in wineries and vineyards, respectively. These compounds have recognized negative effects on yeast growth and fermentative activity which could lead to stuck and sluggish fermentations [74]. The results obtained showed that *S. ludwigii* UTAD17 displayed high resistance to SO_2_ (4 mM), ethanol (12% *v*:*v*^−1^), and copper (2 mM) (Figure S1). The ability to produce biogenic animes which are toxic to humans, [21] was also evaluated. Interestingly, *S. ludwigii* UTAD17 did not present decarboxylase activities responsible for the production of histamine, tyramine and putrescine. On the other hand, *S. ludwigii* UTAD17 exhibited β-glucosidase and β-lyase activities involved in the liberation of terpenes from glycosylated precursors [12] and volatile thiols from cysteinylated precursors [19]. In line with the results obtained, *S. ludwigii* UTAD17 presents important features for a wine yeast starter, since it is able to adjust to winemaking stress, can contribute to the improvement of wine aromatic profile and does not compromise consumers’ health.

### 3.2. Yeast Growth Kinetics and Fermentation Profiles 

To evaluate the performance of *S. ludwigii* UTAD17 in winemaking conditions, fermentations were conducted either in single culture or in consortium with the commercial wine strain *Saccharomyces cerevisiae* QA23, inoculated simultaneously or sequentially, at 72 h. In parallel, a control fermentation was carried out using *Saccharomyces cerevisiae* QA23 in single culture, for comparison.

**Figure 1:**
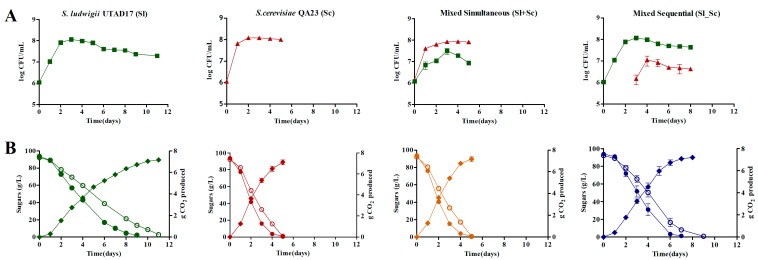
Means ± standard deviations of (**A**) yeast cell counts of *S. ludwigii* UTAD17 (green squares) and *S. cerevisiae* QA23 (red triangles) in single and mixed cultures; and (**B**) Fermentation profiles (diamonds), glucose (filled circles), and fructose (clear circles) concentrations during single and mixed culture trials.

The growth dynamics and fermentation profiles for each single and mixed culture trials are presented in Figure 1. All fermentations were completed, although differences in the total time of fermentation were observed. The *S. ludwigii* UTAD17 showed a sugar uptake preference similar to *S. cerevisiae* strains, consuming glucose more rapidly than fructose. While in pure culture, *S. ludwigii* UTAD17 displayed a significantly lower fermentation rate than *S. cerevisiae*, although it was able to ferment the grape must sugar to dryness (below 4 g L^−1^) within 11 days, six more days than the time required for the high fermenter strain *S. cerevisiae* Lalvin QA23 (Table 1 and Figure 1B). This lower fermentative activity of *S. ludwigii* UTAD17 was not attributable to the differences in the biomass, which is known to have a great influence in determining the fermentation activity [69,75]. Indeed, both species, inoculated at the same amount (1 × 10^6^ cfu·mL^−1^), resumed growth almost immediately after inoculation and, albeit with differences in the growth rate (Figure 1A), achieved similar maximum cell populations—1.2 × 10^8^ cfu·mL^−1^ for *S. cerevisiae* (after 48 h) and 1.1 × 10^8^ cfu·mL^−1^ for *S. ludwigii* UTAD17 (after 72 h). A low fermentative capacity together with a high susceptibility to ethanol is believed to underlie the reduced competitiveness of non-*Saccharomyces* species along wine fermentation [76]. The phenotypic screening performed showed that *S. ludwigii* UTAD17 is able to tolerate up to 12% (*v*:*v*^−1^) of ethanol (Figure S1), which is above the 10.2–10.4% (*v*:*v*^−1^) achieved in the final wines, indicating that the decline in cell viability registered in the later stage of the fermentation should result from other factors. The predicted ORFeome of *S. ludwigii* UTAD17 showed that this species is equipped with enzymes required for ethanol production from glucose, including hexoses transporters, glycolytic enzymes, and alcohol dehydrogenases (Table S1). Recently, genomic sequencing of an *H. guilliermondii* wine strain revealed that one of the key factors contributing to the reduced fermentation ability of this species is the lack of genes for the biosynthesis of thiamine [77], a cofactor of the pyruvate decarboxylase enzyme that is known to play an essential role in determining the regulation of the glycolytic flux [78,79]. This is not apparently the case in *S. ludwigii* UTAD17, since the thiamine-biosynthesis genes could be predicted from the genomic sequence of the UTAD17 strain (Table S1) [67]. Further studies are required to understand the lower fermentation rate exhibited by the *S. ludwigii* UTAD17, in comparison with *S. cerevisae*, one of the possibilities being a low activity of critical glycolytic enzymes, as recently shown to be the case in *H. uvarum* pyruvate kinase [80]. Contrary to that observed for *S. cerevisiae* which almost entirely consumed the nitrogen available in the medium, *S. ludwigii* UTAD17 displayed a preferential consumption of amino acids (PAN) over ammonium and left about 60 mg/L of YAN in the final wine (Table 1). It has been proposed that differences in the efficiency of nitrogen consumption, in general, and in the ability to uptake specific nitrogen sources from the grape-must account for variations in the fermentative activity of *S. cerevisiae* strains [81,82]. In this context, it would be interesting to determine whether the differences observed in the fermentation performances of *S. cerevisiae* and *S. ludwigii* UTAD17 (Figure 1) are the result of differences in their nitrogen uptake capability (Table 1). 

When the two strains were co-inoculated simultaneously (Sc+Sl), a decrease in both strain populations was noted to likely reflect the more competitive environment in terms of space [83] and nutrients, two factors that were previously found to determine yeast–yeast interactions [32,74]. Yet, *S. cerevisiae* dominated over *S. ludwigii* UTAD17, in line with the notion that *S. cerevisiae* is better adapted to grape-juice per se than non-*Saccharomyces* yeasts [84]. Notably, although *S. ludwigii* UTAD17 was able to maintain a substantial viable population throughout these Sc+Sl fermentations, the rate and total time of fermentation was mostly similar, compared to *S. cerevisiae* in a single culture. In the sequential mixed-culture trials (Sl_Sc), *S. ludwigii* UTAD17 initiated fermentation, and *S. cerevisiae* QA23 was later inoculated at 72 h. At this stage, about 40% of the initial sugars were fermented by *S. ludwigii* UTAD17 and the assimilable nitrogen concentration was significantly lowered (Table 1). Contrary to that observed in simultaneous fermentations, in this case, *S. ludwigii* UTAD17 dominated over *S. cerevisiae.* Indeed, growth of the non-*Saccharomyces* strain proceeded as in a single culture, exhibiting a similar growth rate and loss of viability. On the other hand, in these experiments *S. cerevisiae* QA23 growth was limited to a maximum population of about 1.1 × 10^7^ cfu mL^−1^, attained 24 h after its inoculation, most probably due to the low assimilable nitrogen available in the medium. Nevertheless, there was an increase in the fermentation rate and sequential fermentations were successfully completed in eight days, taking 72 h more than *S. cerevisiae* in single culture. Adjusting the YAN levels of the fermenting must at this stage, could be an option to improve the growth of *S. cerevisiae* and the fermentation performance.

### 3.3. Effect of S. ludwigii UTAD17 on Wine Composition and Aroma Profile

The primary physiochemical parameters of the wines obtained are presented in Table 1. The production of ethanol is an essential attribute to define the use of yeasts in the production of fermented beverages. *S. ludwigii* UTAD17 showed a similar efficiency of sugar-to-ethanol conversion to that of *S. cerevisiae* QA23, as the ethanol levels of the final wines, which ranged from 10.2 to 10.4% (*v*:*v*^−1^), were not significantly different. Likewise, no significant differences were found on the amount of SO_2_ formed in each fermentation. Both strains produced up to 20 mg mg L^−1^ and thus they were considered low-sulfite-forming yeasts [85]. Overall, all fermentations resulted in lower levels of acetic acid. Slightly lower levels of acetic acid were found in wines where *S. ludwigii* UTAD17 was involved, as compared to the wines only fermented by *S. cerevisiae* and significantly lower levels of this compound were detected on the sequentially inoculated wines (Table 1). These are promising features since the amount of both metabolites is tightly limited by regulations, might depreciate wine aroma (especially acetic acid) or raise concerns about consumers’ safety (SO_2_) and, thus, they should be kept at the lowest possible levels. The lower acetic acid produced in the fermentations dominated by the non-*Saccharomyces* yeast was accompanied by significantly lower levels of glycerol in all fermentations, suggesting that *S. ludwigii* UTAD17 management strategy for NADH/NAD^+^ recycling and maintenance of redox balance is similar to that of *S. cerevisiae* [86]. This connection between acetic acid and glycerol production is not as clear in other non-*Saccharomyces*. For instance, in single culture fermentations, *Starmerella bacillaris* appears to be a high glycerol and low acetic acid producer [53], while *H. uvarum* seems to be a low glycerol and high acetic acid producer of yeast [87]. 

**Figure 2:**
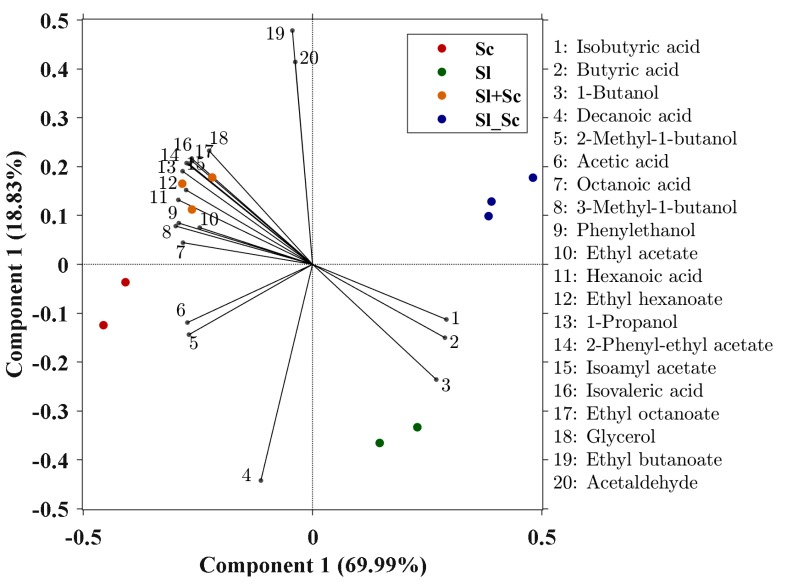
Partial least squares-discriminant analysis (PLS–DA) plot of wines obtained with the different inoculation strategies using volatile and non-volatile compounds that were significantly different among treatments—single-culture of *S. ludwigii* UTAD17(Sl) and *S. cerevisiae* (Sc) or in consortium—mixed simultaneously (Sl+Sc) and sequentially (Sl_Sc).

In order to evaluate how *S. ludwigii* UTAD17 affected the final aroma composition, the different wines were analyzed by gas chromatography. Eighteen yeast-derived aroma compounds were quantified, five alcohols, six acids, four ethyl-esters, two acetates, and one aldehyde (Table 2). Those compounds that were found to be significantly different (*p* < 0.05), along with glycerol and acetic acid (Table 1), were used for Partial Least Squares–Discriminant Analysis (PLS–DA), in order to distinguish the wines obtained with the different inoculation strategies (Figure 2). The first component accounted for 69.99%, while component 2 explained 18.83% of the total variation. Replicate experiments were well grouped on the PLS. A clear separation was observed between the wines whose fermentation were dominated by *S. cerevisiae*, and those governed by *S. ludwigii* UTAD17. Although *S. ludwigii* UTAD17 produced overall significantly lower levels of volatile compounds (Table 2), it should be highlighted that the wines obtained by simultaneous co-inoculation of both strains (Sl+Sc), located in the upper-left quadrant (Figure 2), were characterized by a greater diversity of flavors and complexity, as compared with those fermented by *S. cerevisiae* alone. Overall, fermentations conducted by *S. ludwigii* UTAD17 resulted in wines characterized by higher levels of 1-butanol and butyric and isobutyric acids, which were found to be 2 to 3 times higher than that in Sc and Sc+Sl wines (Table 2). 1-butanol and the short-chain fatty acid, butyric acid, are synthetized from 2-ketovalerate following decarboxylation to the aldehyde precursor, butyraldehyde, which is either reduced or oxidized, respectively [88]. In this study we could only speculate that the high levels of both compounds produced by *S. ludwigii* UTAD17 could result from either an excess of the intermediate α-keto acid or the use of this redox duality of the last steps of the Ehrlich pathway to help maintain the redox balance of the cell [89].

Previous studies have put aside the use of *S. ludwigii* in the winemaking industry, given the large amounts of ethyl acetate and acetaldehyde produced by these strains [7,8,61]. Notably, the *S. ludwigii* UTAD17 strain is, apparently, a low producer of both compounds as the levels detected were significantly inferior to those obtained in both Sc and Sl+Sc fermentations. Additionally, none-to-low levels of acetate and ethyl esters, which are responsible for the pleasant fruity and floral bouquet of wines, were found to be produced by *S. ludwigii* UTAD17. The predicted UTAD17 ORFeome did not include proteins similar to the *S. cerevisiae* acetyl transferases ScATF1, ScATF2, and ScAYT1, required for synthesis of acetate esters (Table S1). As such, the minor amounts of these compounds found on Sl_Sc fermentations was likely attributable to *S. cerevisiae* QA23 activity. Contrarily, we found that *S. ludwigii* UTAD17 harbors proteins similar to *S. cerevisiae* ethanol acyl-coA transferases ScEHT1 and ScEEB1, responsible for ethyl ester synthesis through the condensation between ethanol and fatty acyl-CoA [90], and ScIAH1 involved in esters hydrolysis [91]. In this case, the limited production of ethyl esters by *S. ludwigii* UTAD17 might result from differences in the activity levels of these enzymes. A more in-depth analysis of this genomic information is being undertaken that will shed light on the molecular foundations underlying some of the intriguing physiological traits of this yeast such as high SO_2_ resistance, lower fermentative power, and wine aroma. From a practical point of view, this information will be very useful for a rational application of this strain, depending on the winemaking conditions

Overall, the above-described results gave good insights on the potential use of *S. ludwigii* UTAD17 in winemaking. Given the low fruity/estery nature of this strain, it could be a good option to obtain wines with a greater expression of grape varietal characteristics. Nowadays, winemakers make use of a blend of wines fermented with different yeast strains/species or different grape varieties to create wines tailored to meet consumer expectations. Following this line of winemaking, and considering the trend of the use of non-*Saccharomyces* yeasts to obtain wines with distinct aroma profiles, it should be underlined that the aromatic characteristics of *S. ludwigii*, UTAD17, which greatly differs from the traditional *S. cerevisiae*, might confer a peculiar imprint onto the final product. Nevertheless, our results warrant further studies to evaluate whether the observed differences in chemical composition can be perceived during sensory evaluation.

## Figures and Tables

**Table 1:** Physicochemical composition of initial grape-must and wines obtained by single-cultures of *S. ludwigii* UTAD17 (Sl) and *S. cerevisiae* QA23 (Sc) or in consortium—mixed simultaneously (Sl+Sc) and sequentially (Sl_Sc) in natural grape-juice of *Vitis vinifera* L. cv. Touriga Nacional at 25 °C under static conditions.

Compound	Grape-Must	Sl	Sc	Sl+Sc	Sl_Sc
Sugars (g L^−1^)	182.140 ± 3.62	2.273 ± 0.733 ^a^	0.328 ± 0.284 ^b^	0.190 ± 0.242 ^b^	0.018 ± 0.009 ^b^
Ethanol (% *v*:*v*^−1^)	-	10.195 ± 0.194 ^a^	10.391 ± 0.025 ^a^	10.201 ± 0.077 ^a^	10.355 ± 0.271 ^a^
Glycerol (g L^−1^)	-	6.279 ± 0.024 ^c^	7.671 ± 0.060 ^ab^	7.659 ± 0.302 ^a^	6.800 ± 0.658 ^bc^
Acetic Acid (g L^−1^)	-	0.138 ± 0.004 ^bc^	0.170 ± 0.018 ^a^	0.149 ± 0.004 ^ab^	0.120 ± 0.011 ^c^
Titratable Acidity (g L^−1^)	8.010 ± 0.350	8.100 ± 0.120 ^ab^	7.980 ± 0.000 ^a^	8.390 ± 0.010 ^a^	7.690 ± 0.130 ^b^
Total SO_2_ (mg L^−1^)	-	14.830 ± 0.430 ^a^	15.300 ± 0.820 ^a^	15.360 ± 0.000 ^a^	16.380 ± 3.020 ^a^
pH	2.990 ± 0.011	2.957 ± 0.003 ^a^	2.926 ± 0.031 ^b^	2.961 ± 0.000 ^a^	2.956 ± 0.004 ^a^
YAN (mg L^−1^)	196.869 ± 2.339	61.214 ± 5.029 ^a^	5.250 ± 0.354 ^c^	4.000 ± 0.000 ^c^	22.594 ± 6.731 ^b^
YAN(_72 h)_ (mg L^−1^)	-	79.584 ± 1.996 ^a^	12.250 ± 3.889 ^b^	13.168 ± 0.289 ^b^	68.418 ± 19.340 ^a^
PAN (mg L^−1^)	92.000 ± 9.899	20.500 ± 2.121 ^a^	5.250 ± 0.354 ^b^	4.000 ± 0.000 ^b^	19.167 ± 4.537 ^a^
PAN_(72 h)_ (mg L^−1^)	-	29.000 ± 1.414 ^a^	12.250 ± 3.889 ^b^	13.168 ± 0.289 ^b^	26.333 ± 4.646 ^a^
NH_4_ (mg L^−1^)	127.500 ± 9.192	49.500 ± 3.536 ^a^	nd ^c^	nd ^c^	4.170 ± 2.843 ^b^
NH_4(72 h)_ (mg L^−1^)	-	61.500 ± 0.707 ^a^	nd ^b^	nd ^b^	51.167 ± 17.905 ^a^
Sugars 72 h (g L^−1^)	-	109.252 ± 1.555 ^a^	41.174 ± 0.246 ^b^	42.883 ± 2.115 ^b^	101.200 ± 11.232 ^a^
M_ax_FR (g of CO_2 V_ h^−1^).	-	0.051 ± 0.004 ^b^	0.100 ± 0.005 ^a^	0.098 ± 0.002 ^a^	0.058 ± 0.006 ^b^
FP	-	0.014 ± 0.001 ^a,b^	0.017 ± 0.002 ^a^	0.015 ± 0.001 ^a^	0.011 ± 0.002 ^b^

Data are expressed as triplicate means for mixed trials and duplicate means for single culture trials ± standard deviations. Values in the same row with different superscript letters are significantly different (*p* < 0.05). YAN—yeast assimilable nitrogen. PAN—primary amino nitrogen. MaxFR—maximum fermentation rate. FP—fermentation purity (acetic acid (g L^−1^)/ ethanol (% *v*:*v*^−1^)). nd—not detected. -: not measured.

**Table 2:** Concentration of volatile compounds detected and quantified by Gas Chromatography equipped with Flame ionization Detector (GC–FID) in wine obtained by a single-culture of *S. ludwigii* UTAD17(Sl) and *S. cerevisiae* (Sc) or in consortium—mixed simultaneous (Sl+Sc) and sequential (Sl_Sc), in natural grape-juice of *Vitis vinifera* L. cv. Touriga Nacional at 25 °C, under static conditions.

Compound (mg L^−1^)	Sl	Sc	Sl+Sc	Sl_Sc	OT (mg/L)	OD
**Alcohols**						
1-propanol	14.315 ± 0.402 ^c^	44.383 ± 1.298 ^a^	43.985 ± 0.516 ^a^	17.934 ± 1.557 ^b^	306.000	Alcohol, ripe fruit
1-butanol	35.428 ± 1.045 ^a^	15.040 ± 0.220 ^c^	15.335 ± 1.428 ^c^	30.792 ± 0.316 ^b^	150.000	Medicinal
2-Methyl-1-butanol	17.882 ± 1.033 ^ab^	19.040 ± 0.721 ^a^	18.966 ± 1.087 ^a^	15.123 ± 0.436 ^b^	30.000	Alcohol, nail polish
3-Methyl-1-butanol	74.963 ± 6.858 ^b^	102.984 ± 3.465 ^a^	101.176 ± 1.794 ^a^	69.184 ± 1.217 ^b^	30.000	Whiskey, nail polish
2-Phenylethanol	18.945 ± 0.728 ^b^	27.040 ± 1.881 ^a^	26.997 ± 1.398 ^a^	16.869 ± 1.015 ^b^	14.000	Rose, honey
**⅀**	161.534 ± 8.609 ^b^	208.486 ± 3.824 ^a^	206.459 ± 6.122 ^a^	149.962 ± 4.092 ^b^		
**Acetate Esters**						
Phenylethyl Acetate	nd^b^	0.181 ± 0.011 ^a^	0.177 ± 0.024 ^a^	nd^b^	0.250	Flowery
Isoamyl Acetate	nd^c^	0.807 ± 0.106 ^a^	0.725 ± 0.155 ^a^	0.171 ± 0.120 ^b^	0.030	Banana
**⅀**	nd^c^	0.988 ± 0.117 ^a^	0.902 ± 0.163 ^a^	0.171 ± 0.120 ^b^		
**Ethyl Esters**						
Ethyl Acetate	32.679 ± 6.895 ^b^	40.995 ± 0.393 ^ab^	47.392 ± 7.071 ^a^	28.387 ± 5.742 ^b^	7.500	Fruity, vinegar, nail polish, acetic
Ethyl Butanoate	nd^b^	0.604 ± 0.182 ^a^	0.630 ± 0.066 ^a^	0.669 ± 0.118 ^a^	0.020	Apple, strawberry, fruity
Ethyl Hexanoate	nd^d^	0.301 ± 0.012 ^a^	0.231 ± 0.024 ^b^	0.082 ± 0.020 ^c^	0.005	Green apple, fruity
Ethyl Octanoate	nd^b^	0.312 ± 0.046 ^a^	0.410 ± 0.080 ^a^	nd^b^	0.002	Pear, fruity
**⅀ (except ethyl acetate)**	nd^c^	1.216 ± 0.149 ^a^	1.271 ± 0.160 ^a^	0.752 ± 0.132 ^b^		
**Fatty Acids**						
Isobutyric Acid	2.430 ± 0.072 ^a^	1.077 ± 0.194 ^b^	1.242 ± 0.205 ^b^	2.494 ± 0.172 ^a^	2.300	Fatty
Butyric Acid	1.892 ± 0.074 ^a^	0.631 ± 0.089 ^b^	0.726 ± 0.056 ^b^	1.880 ± 0.074 ^a^	10.000	Fatty, rancid
Isovaleric Acid	nd^b^	0.231 ± 0.040 ^a^	0.251 ± 0.045 ^a^	nd^b^	0.033	Fatty, rancid
Hexanoic Acid	0.591 ± 0.023 ^c^	1.435 ± 0.064 ^a^	1.350 ± 0.027 ^b^	0.558 ± 0.034 ^c^	0.420	Cheese, fatty
Octanoic Acid	0.727 ± 0.175 ^c^	2.383 ± 0.060 ^a^	1.559 ± 0.127 ^b^	0.710 ± 0.145 ^c^	0.500	Fatty, unpleasant
Decanoic Acid	0.222 ± 0.005 ^a^	0.224 ± 0.031 ^a^	nd ^b^	nd ^b^	1.000	Fat, rancid
**⅀**	5.861 ± 0.293 ^a^	5.980 ±0.475 ^a^	5.127 ± 0.145 ^b^	5.642 ± 0.329 ^ab^		
**Acetaldehyde**	20.279 ± 0.472 ^b^	25.328 ± 1.400 ^a^	25.661 ± 1.500 ^a^	25.944 ± 2.672 ^a^	10.000	Sherry, nutty, bruised apple

Data are expressed as means ± standard deviations resulting from triplicate experiments for mixed trials and duplicates for single culture trials. OT—odor threshold; OD—odor descriptions. Odor thresholds and odor descriptions can be found in the literature [92,93,94,95,96,97,98]. Values in the same row with different superscript letter are significantly different (*p* < 0.05). nd—not detected.

## Supplementary Materials

The following are available online at https://www.mdpi.com/2076-2607/7/11/478/s1, Figure S1: Phenotypic profile of the *S. ludwigii* UTAD17; Table S1: Comparative analysis of the predicted *S. ludwigii* UTAD17 ORFeome with sets of proteins of *S. cerevisiae* S288c
