# Supplementary material for: Characterizing the Potential of the Non-Conventional Yeast Saccharomycodes ludwigii UTAD17 in Winemaking
Source: Microorganisms. 2019 Oct 23;7(11):478. doi: 10.3390/microorganisms7110478 (PMC6920815; doi:10.3390/microorganisms7110478)
Supplement: Supplementary file 1 [file microorganisms-07-00478-s001.zip › Figure S1.pdf]

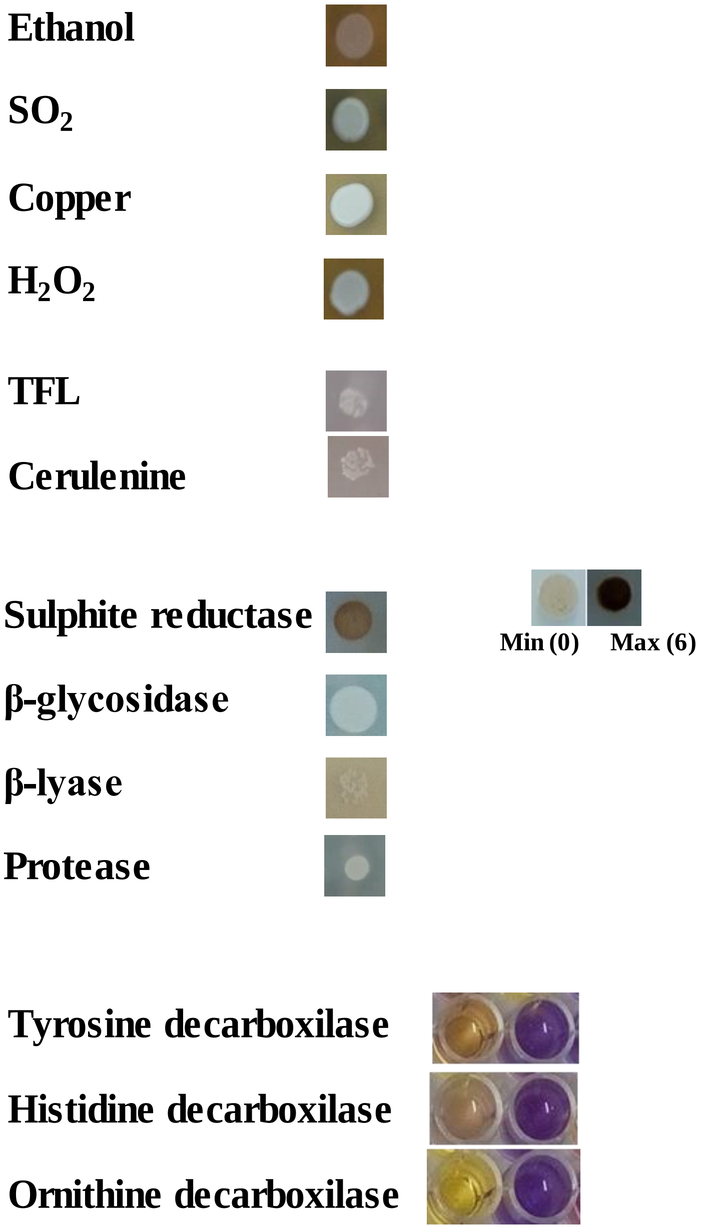

Fig. S1 - Phenotypic profile of the *S. ludwigii* UTAD17.

Growth of *S. ludwigii* UTAD17 strain under the highest dose of each stress agent tested is presented (ethanol 12% (v/v); SO_2 – 4mM; copper – 2mM; H_2O_2 – 1mM; cerulenin – 6 µM and TFL – 0.6 mM). Enzymatic activities were associated with the ability to grow in the different culture media with the specific substrates, as described in Material and Methods. For sulphite reductase, *S.ludwigii* UTAD17 colony colour on BIGGY agar was scored as 3, in a scale ranging from 0 (white) to 6 (brown to near-black), as illustrated. For protease activity, the absence of a clear zone around the colony was associated to the null activity of *S. ludwigii* UTAD17. The inability of *S. ludwigii* UTAD17 to produce biogenic amines, histamine, tyramine and putrescine, was detected by the absence of decarboxylation of correspondent amino acid, histidine, tyrosine or ornithine (the medium became yellowish), contrasting to the purple coloration associated with decarboxylation reactions.
