# Supplementary material for: Characterizing the Potential of the Non-Conventional Yeast Saccharomycodes ludwigii UTAD17 in Winemaking
Source: Microorganisms. 2019 Oct 23;7(11):478. doi: 10.3390/microorganisms7110478 (PMC6920815; doi:10.3390/microorganisms7110478)
Supplement: Supplementary file 1 [file microorganisms-07-00478-s001.zip › Table S1.pdf]

Table S1: Comparative analysis of the predicted *S. ludwigii* UTAD17 ORFeome with the sets of proteins of *S. cerevisiae* S288c.

S. cerevisiae gene	Protein Function	*S. ludwigii* UTAD17 orthologous ORF name
HXT1/HXT6	Hexose Transporter (low-affinity)	**SCODWIG_02272**/**SCODWIG_01057**/SCODWIG_03801/SCODWIG_03974/SCODWIG_03563/SCODWIG_04015
HXT2	Hexose Transporter (high-affinity)	**SCODWIG_02272**/**SCODWIG_01057**/SCODWIG_03801/SCODWIG_03974/SCODWIG_03563/SCODWIG_04015/SCODWIG_03454
HXT3/HXT5	Hexose Transporter (low-affinity)	**SCODWIG_01057**/**SCODWIG_02272**/SCODWIG_03801/SCODWIG_03974/SCODWIG_03563/SCODWIG_04015/SCODWIG_03454
HXT4/HXT7	Hexose Transporter (high-affinity)	**SCODWIG_01057**/**SCODWIG_02272**/SCODWIG_03801/SCODWIG_03974/SCODWIG_03563/SCODWIG_04015/SCODWIG_03454
HXT8	Hexose Transporter (unknown function)	**SCODWIG_02272**/**SCODWIG_01057**/SCODWIG_03801/SCODWIG_03974/SCODWIG_03563/SCODWIG_04015/SCODWIG_03454
HXT9	Hexose Transporter (putative)	**SCODWIG_01057**/**SCODWIG_02272**/SCODWIG_03801/SCODWIG_03974/SCODWIG_03563/SCODWIG_04015/SCODWIG_03454
HXT10	Hexose Transporter (putative)	**SCODWIG_02272**/**SCODWIG_01057**/SCODWIG_03801/SCODWIG_03974/SCODWIG_03563/SCODWIG_04015/SCODWIG_03454
HXT11	Hexose Transporter	**SCODWIG_01057**/**SCODWIG_02272**/SCODWIG_03801/SCODWIG_03974/SCODWIG_03563/SCODWIG_04015
HXT13/HXT17	Hexose Transporter (putative transmembrane polyol transporter)	**SCODWIG_02272**/**SCODWIG_01057**/SCODWIG_03801/SCODWIG_03974/SCODWIG_03563/SCODWIG_04015
HXT14	Hexose Transporter	SCODWIG_03563/SCODWIG_02272/SCODWIG_01057/SCODWIG_03801/SCODWIG_03974/SCODWIG_04015
HXT15	Hexose Transporter (putative transmembrane polyol transporter)	**SCODWIG_02272**/**SCODWIG_01057**/SCODWIG_03801/SCODWIG_03974/SCODWIG_03563/SCODWIG_03454/SCODWIG_04015
HXT16	Hexose Transporter (putative transmembrane polyol transporter)	**SCODWIG_02272**/SCODWIG_03801/SCODWIG_03974/SCODWIG_03563/SCODWIG_03454/SCODWIG_04015
GAL2	Galactose Permease	**SCODWIG_02272**/**SCODWIG_01057**/SCODWIG_03801/SCODWIG_03974/SCODWIG_03563/SCODWIG_04015/SCODWIG_03454
HXK1/HXK2	Hexokinase isoenzyme	**SCODWIG_02966/**SCODWIG_01735
GLK1/EMI2	Glucokinase	**SCODWIG_01735/**SCODWIG_02966
PGI1	Phosphoglucose isomerase	**SCODWIG_01883**
PFK1	Phosphofructokinase (alpha subunit)	**SCODWIG_02053**
PFK2	Phosphofructokinase (beta subunit)	**SCODWIG_01491**
FBA1	Fructose 1,6-bisphosphate aldolase	**SCODWIG_00116**
TPI1	Triose-Phosphate Isomerase	**SCODWIG_00702**
TDH1	Triose-Phosphate Dehydrogenase isoenzyme 1	**SCODWIG_02663/SCODWIG_02233/**SCODWIG_00205

TDH2/TDH3	Triose-Phosphate Dehydrogenase isoenzyme 2	**SCODWIG_02233/SCODWIG_02663/**SCODWIG_00205
PGK1	3-PhosphoGlycerate Kinase	**SCODWIG_01455**
GPH1	Glycogen Phosphorylase	**SCODWIG_02626**
ENO1/ENO2	Phosphopyruvate hydratase	**SCODWIG_00273**
PYK12/CDC19	Pyruvate kinase	**SCODWIG_03273**
PDC5	Pyruvate decarboxylase (minor isoform)	**SCODWIG_02550/SCODWIG_03821**
PDC6	Pyruvate decarboxylase (minor isoform)	**SCODWIG_02550/SCODWIG_03821**
PDC1	Pyruvate decarboxylase (major isoform)	**SCODWIG_02550/SCODWIG_03821**
ADH5/ADH1	Alcohol dehydrogenase	**SCODWIG_02765/SCODWIG_01877/**SCODWIG_00922/SCODWIG_04004/ SCODWIG_01402
ADH4	Alcohol dehydrogenase (zinc-dependent)	-
ADH3	Alcohol dehydrogenase(mitochondrial)	**SCODWIG_01877/SCODWIG_02765/**SCODWIG_04004/SCODWIG_00922
ADH2	Alcohol dehydrogenase (glucose-repressible)	**SCODWIG_02765/**SCODWIG_01877/SCODWIG_04004/SCODWIG_00922
ADH6	Alcohol dehydrogenase (NADPH-dependent)	SCODWIG_02296/SCODWIG_01877
ADH7	Alcohol dehydrogenase (NADPH-dependent)	SCODWIG_02296
THI13	HMP-P precursor synthesis	-
THI20	HMP kinase and HMP-P kinase	SCODWIG_03914/SCODWIG_03996/SCODWIG_04014
THI21	HMP kinase and HMP-P kinase	SCODWIG_03914/SCODWIG_03996/SCODWIG_04014
THI4	Thiazole synthase	SCODWIG_02921
THI6	Thiamine-phosphate diphosphorylase and hydroxyethylthiazole kinase	**SCODWIG_02681**
THI80	Thiamine pyrophosphokinase	SCODWIG_03709
THI10/THI17	Thiamine transporter	**SCODWIG_03263/SCODWIG_02398/SCODWIG_03795/**SCODWIG_00195/ SCODWIG_03794
ATF1	Alcohol acetyltransferase	-
ATF2	Alcohol acetyltransferase	-
AYT1	Acetyltransferase	-
EEB1/EHT1	Acyl-coenzymeA:ethanol O-acyltransferase	SCODWIG_03242/SCODWIG_00846
MGL2	Monoacylglycerol and triacylglycerol lipase (minor Acyl-transferase activity)	SCODWIG_00846
IAH1	Isoamyl acetate-hydrolyzing esterase	SCODWIG_03553

S. ludwigii UTAD17 proteins were considered similar to those present in *S. cerevisiae* S288c when the associated alignment had a respective e-value below e^{-20} and a minimum identity of 30%. ORF's with an e-value of 0.0 are marked in bold.
